# Cigarette and E-Cigarettes Dual Users, Exclusive Users and COVID-19: Findings from Four UK Birth Cohort Studies

**DOI:** 10.3390/ijerph18083935

**Published:** 2021-04-08

**Authors:** Daniel Tzu-Hsuan Chen, Christina N. Kyriakos

**Affiliations:** Public Health Policy Evaluation Unit, School of Public Health, Imperial College London, London W6 8RP, UK; c.kyriakos20@imperial.ac.uk

**Keywords:** COVID-19, smoking, e-cigarettes, birth cohort, United Kingdom, poly-tobacco, dual use

## Abstract

Introduction: The relationship between current cigarette and electronic cigarette (e-cigarette) dual use, exclusive use and COVID-19-related measures are still unclear. This study aims to assess the association between different tobacco use patterns and coronavirus disease 2019 (COVID-19) symptoms, testing, self-reported infection and social distancing behaviors in the United Kingdom (UK). Methods: Data come from the first wave of the Centre for Longitudinal Studies (CLS) COVID-19 survey, comprising four birth cohorts (N = 13,077, aged 20–63 years) surveyed between 2 to 31 May 2020, during the COVID-19 pandemic. Sociodemographic characteristics and COVID-19-related outcomes (symptoms, testing, diagnosis and social distancing behaviors) were compared across different product user groups (non-users, exclusive cigarette users, exclusive e-cigarettes users and dual use) using Cochran–Mantel–Haenszel χ2 test. Multivariable logistic regression models were used to explore associations between COVID-19-related outcomes and different smoking patterns. Results: Across all four cohorts, 12.6% and 4.9% of the respondents were current exclusive cigarette and e-cigarette users, respectively, with approximately 3% of the respondents being dual users. Significant differences in prevalence were observed between different tobacco use patterns and COVID-19 symptoms (*p* = 0.02), self-reported infection (*p* = 0.04) and social distancing behaviors (*p* < 0.001). Current cigarette and e-cigarette dual use was associated with 2.15-fold higher odds for reporting COVID-19 infection (aOR = 2.15; CI [1.15–4.05]). Compliance of social distancing behaviors were the lowest for current dual users (aOR = 0.58; CI [0.41–0.83]) and exclusive cigarette users (aOR = 0.72; CI [0.63–0.92]). Conclusions: The findings highlight dual users’ higher prevalence of having COVID-19 symptoms, infection and incompliance of social distancing behaviors. Self-reported infection was associated with dual product use; dual users and exclusive cigarette users were linked to poor adherence to social distancing behaviors. Smoking cessation support and further monitoring on multiple tobacco use among these populations should be reinforced as preventive measures to tackle the pandemic.

## 1. Introduction

Smoking is a leading cause of premature death and is a well-established risk factor for a myriad of acute and chronic diseases that affect cardiovascular and respiratory systems [1]. The coronavirus 2019 (COVID-19) is an infectious disease that attacks the respiratory systems, while the damage caused to the lungs by smoking makes people more susceptible to viral and bacterial infections [2]. Therefore, the current COVID-19 pandemic has spurred inquiry about the impact of smoking on COVID-19 outcomes.

Results from systematic reviews and meta-analyses of existing studies suggest an association between smoking and a greater risk of COVID-19 disease progression, severity, and mortality [3,4,5,6,7]. While it has been postulated that smokers are at reduced risk of infection compared to the general population, the methodological quality of this data has been questioned [8].

The results seen in smokers further raise speculations of whether this also applies to people who use e-cigarettes, vaping products or dual users of both cigarettes and electronic cigarette (e-cigarette) [9]. Sufficient evidence shows using e-cigarettes, or vaping, is harmful to lung function [10,11]. Both cigarette and e-cigarette use damage the respiratory system [10,12], potentially increasing the risk of experiencing COVID-19-related measures, such as symptoms and diagnosis. This poses an alarming risk for dual users as they might suffer elevated health risks from the use of use multiple tobacco products and increase their susceptibility to infection and result in worse prognosis once infected [9,13,14].

A recent cross-sectional study among a national sample of US adolescents and young adults found that dual users of both cigarettes and e-cigarettes within the past 30 days were more likely to report experiencing COVID-19-related symptoms [15]. However, concerns about the data reliability and the validity of the results have been raised due to issues in response bias and population weighting [16,17,18]. Moreover, there is currently limited research examining electronic cigarette (e-cigarette) use and use of both cigarettes and e-cigarettes (dual use) in relation to COVID-19.

However, the relationship between dual tobacco product use and risk of COVID-19 infection is less clear [13]. The aim of the current study was to assess the association between different tobacco product user groups (non-users, exclusive cigarette users, exclusive e-cigarettes users and dual use) and COVID-19 symptoms, testing, self-reported infection and social distancing behaviors using a nationally representative birth cohort of the UK population.

## 2. Methods

### 2.1. Study Design

Data come from Wave 1 of the University College London Centre for Longitudinal Studies (CLS) COVID-19 study, a series of surveys following large nationally representative cohorts of people on COVID-19-related outcomes during the COVID-19 pandemic. The CLS COVID-19 survey was administered online to participants across four nationally representative cohorts, each representing a different generation of the UK population. The details of the survey design, recruitment procedure, and survey process have been described elsewhere [19]. Briefly, the survey was issued to 38,727 individuals across all cohorts, with around 13,163 of those invited responded, achieving a response rate of 34%. The wave 1 survey took place between 2 to 31 May 2020 at the height of lockdown and social distancing restrictions in the UK.

The analytic sample of the current study include data from four cohorts managed by CLS: The Millennium Cohort Study, born in 2000–2002, aged 18–20 (n = 2403); Next Steps, born in 1989–1990, aged 31 (n = 1780); the 1970 British Cohort Study, born in 1970 aged 51 (n = 4022); and the National Child Development Study, born in 1958, aged 63 (n = 4872). Overall, the sample included 13,077 respondents aged 20–63 years old.

### 2.2. Measures

The respondents self-reported their sociodemographic characteristics (gender, socioeconomic class, self-rated health, and chronic illness), and current tobacco use pattern (non-smoker, exclusive cigarette user, exclusive e-cigarette user, or dual user) at the time of the survey. Cigarette/e-cigarette use, or dual use were assessed by reporting current occasional or daily use of cigarettes and/or electronic cigarettes.

The socioeconomic class was categorized into high, intermediate, and the lower three distinctive groups, according to the respondents’ socioeconomic status prior to the COVID-19 pandemic (so that is not affected by recent job loss or lockdown restrictions), using the 2010 National Statistics Socioeconomic Analytical Classes (NS-SEC) [20]. Responses that were not applicable by the NS-SEC were included in the “not classifiable” category in the analysis. These include those who never worked or were long-term unemployed, such as being a student or under retirement. Self-rated health was reported on an ordinal scale with five response options ranging from excellent to poor. Pre-existing conditions were indicated by whether or not the respondent suffered from a long-standing illness, including cancer, AIDS/HIV, asthma, Chronic Obstructive Pulmonary Disease (COPD), cystic fibrosis, bronchitis, diabetes, high blood pressure, heart diseases, mental, neurological disorders, musculoskeletal and hearing problems. Binary responses of COVID-19-related measures of whether the respondents have experienced any newly developed COVID-19 symptoms during the past two weeks (the presence of any classic symptoms, such as fever, persistent dry cough, shortness of breath, and loss of smell and taste vs. no such symptoms), being tested or self-reported COVID-19 infection at the time of the survey were asked. As, during the early stage of the pandemic, there was a limited available capacity for testing, most of the participants did their testing in a hospital setting. For responses to COVID-19 infection, participants were asked: Do you think that you have or have had Coronavirus (COVID-19)? with response options (a) Yes, confirmed by a positive lab test. (b) Yes, based on strong personal suspicion or medical advice. (c) Unsure and (d) No. Those who replied (a) or (b) were coded as having self-reported COVID-19 infection. Perceived compliance with social distancing during the surveyed period was measured by asking the participants: “On a scale from 0 to 10, where 0 means that you are ‘not complying at all’ and 10 means you are ‘fully complying’, how much would you say you are complying with the guidelines?”. The extent of compliance to social distancing guidelines were then dichotomised into a binary variable of either fully compliant (score of 10) or not fully compliant (score of 0 to 9).

### 2.3. Statistical Analysis

We compared different tobacco use patterns by participant characteristics and COVID-19-related measures within and across cohorts using the Pearson’s χ2 and Cochran–Mantel–Haenszel χ2 test, respectively. Multivariable logistic regression models accounting for sample survey weights were used to explore associations between COVID-19-related measures and different smoking patterns. The primary sampling unit and strata were also accounted for by the stratified survey designs within cohorts. Models were adjusted for sex, socioeconomic class, self-rated health, chronic illness and cohorts. Adjustment was made for survey non-response weight calculated by the CLS team [19] to reduce biases due to low response rates. We used STATA version 16.0 to obtain weighted point estimates (proportions from χ2 tests and adjusted odds ratios [aORs] from multivariable logistic regression models) and 95% confidence intervals (CIs). The svy: command was used to perform the analyses of complex survey data. For all analyses, the bilateral significance level was set to 0.05.

## 3. Results

Table 1 presents the weighted percentages of participant characteristics and COVID-19–related measures by different tobacco use patterns across four cohorts. Overall, 12.6% of the respondents were current exclusive cigarette users, 4.9% were exclusive e-cigarette users, and 3% were dual users. Prevalence of dual use was highest among the youngest Millennium Cohort, born in 2000–2002 (4.7%); while the British Cohort Study, born in 1970, had the highest prevalence of exclusive cigarette (15.2%) and e-cigarette use (6.5%), respectively.

Among the overall sample, all participant characteristics varied significantly by tobacco use patterns (*p* < 0.05). A higher proportion of dual users were male (54.7%) compared to females (45.3%). Among those with reported socioeconomic class, the proportions of dual users were found to be higher among those with lower socioeconomic class (34.9%) compared to those with higher socioeconomic class (19.1%). A higher percentage of non-users self-rated their health as “excellent” (15.9%) compared to all cigarette and e-cigarette user groups; whereas the proportion of those reported “poor” health were highest among exclusive cigarette users (5.0%) and dual users (3.8%). Compared to non-users (48.6%), exclusive cigarette users, e-cigarette users and dual users reported a higher prevalence of chronic illness (57.1%, 58.9% and 50.2% respectively).

Significant differences were also observed between different tobacco use patterns and COVID-19 symptoms (*p* = 0.02), diagnosis (*p* = 0.04) and social distancing behaviors (*p* < 0.001), with the proportion of dual users higher than that of other smoking pattern groups across all cohorts. In the overall sample, dual users had the highest percentage reporting COVID-19 infection (14.8%) and COVID-19-related symptoms (13.2%) in the past two weeks, compared to all other tobacco use groups. Reported adherence to social distancing was significantly lower among dual users (42.8%) and cigarette users (47.9%, e-cigarette users (50.9%), as compared to non-users (56.9%).

Table 2 shows results from the multivariable logistic regression models on the association between COVID-related outcomes and product user type. Dual users and exclusive cigarette users had lower odds of adhering to social distancing behaviors compared to non-users (aOR = 0.58; CI [0.41–0.83] and aOR = 0.72; CI [0.63–0.92]). No association was observed for experiencing COVID-19 symptoms within the past two weeks or receiving a COVID-19 test by user group. However, current cigarette and e-cigarette dual users had 2.15 greater odds of having a self-reported COVID-19 infection (CI [1.15–4.05]) compared to non-smokers.

## 4. Discussion

Results from four nationally representative birth cohorts provided weighted estimates of current tobacco use patterns (exclusive use of cigarettes or e-cigarettes, or dual use) and COVID-19-related measures during the early period of the pandemic. We further assessed the association between different tobacco use patterns and COVID-19 symptoms, testing, self-reported infection and social distancing behaviors in the UK.

There were significant differences between tobacco use patterns and all participant characteristics across all cohorts. The overall prevalence of current dual users was higher among male, having lower socioeconomic class, poorer self-rated health and higher prevalence of pre-existing condition compared to non-users. Additionally, similar to previous findings, tobacco users were found to have significantly higher percentages for experiencing COVID-19 symptoms, self-reported COVID-19 infection and lower adherence to COVID-19 protective behaviors [15,21]. However, the proportion of those who received a COVID-19 test did not differ between different tobacco use patterns. This may be due to the fact that the estimations presented were weighted characteristics from the probability sample of cohorts that were more likely to reflect the proportions of testing taken in the general population at the early stage of the pandemic. For this reason, the presumption that smokers/tobacco users may have received more COVID-19 testing [22] is not supported in the current study.

The observed gradients in occupational social class and self-reported health among tobacco users suggest the inequality of smoking during the pandemic. This may be of particular relevance in the context of COVID-19 and dual product user, as evidence suggests increased vulnerability to the virus among tobacco product users [4,5] and those with lower social class and health status are at higher risk to respiratory infections and increased probability of having pre-existing conditions [2,23] that could make COVID-19 infections more susceptible to severe symptoms, thus leading to increased mortality [2].

Furthermore, multivariable logistic regression models revealed that dual users were associated with over two-fold higher odds of confirmed or suspected COVID-19 diagnosis, and decreased odds of compliance of social distancing by a quarter for exclusive cigarette users and a half for dual users, compared to non-tobacco users. Heightened odds of self-reported COVID-19 infection among dual tobacco users could be due to the fact that dual users were exposed to elevated levels of nicotine and toxicants via cigarette smoking and vaping, which adversely affects lung function [13,14], thus leading to a significant increase of susceptibility to COVID-19 infection compared to single product users. While we only found a significant association of self-reported COVID-19 infection for dual users, other studies have found an association between exclusive cigarette or e-cigarette use, respectively, with more severe COVID-19 progression [6,7,12].

Assessments regarding tobacco use status in the emerging pandemic is challenging as the methodology used to classify smoking status in studies may not be nuanced enough to capture status change between recent quitters/former smokers and non-smokers. Thus, any former smoking/vaping behavior, which could be associated with COVID-related outcomes, would not have been captured by the measurement of current smoking/vaping status.

Our findings that smokers and dual users self-reported lower adherence to social distancing suggest that being a smoker can make social distancing more challenging [21]. This association may be reflective of the social aspect of tobacco product use. Smoking behaviors are strongly related to social circumstances such as peer smoking, cigarette and device sharing [24,25], which is deemed as a means of social networking and maintaining relationships for smokers [26]. However, such behaviors not only pose risks in maintaining social distancing, the hand-to-mouth movements of smoking/vaping, and device sharing could potentially increase exposure to the COVID-19 [13]. Lower adherence to social distancing observed among smokers may also be explained by smokers having greater inclination for engaging in other health risk behaviors [27] and exhibiting impulsivity-related traits, such as a lack of premeditation, lack of perseverance, sensation seeking, as well as both negative and positive urgency [28]. The observed association may also be related to socioeconomic and occupational circumstances. While we did control for socioeconomic status, smokers and dual users may be disproportionately essential workers who cannot stay home and therefore may be unable to social distance as effectively.

There is limited and contradictory evidence on the association of tobacco use with adverse outcomes of COVID-19 infection [8]. Our study further supports associations between dual cigarette and e-cigarette use and COVID-19 infection from nationally-representative cohorts of the UK population. The analytical methods used and the population-based survey estimates reported in the current study potentially minimizes the controversies of data reliability and responsiveness raised within previous studies [16,17,18]. Despite the large sample size and similar effects found across four different birth cohorts, this study has several limitations. A limitation of the study is missing data due to low response rates. While all the analyses have been adjusted for survey non-response weight to reduce biases due to low response rates, there is a possibility that unobserved variables of missing data may still influence results. Owing to the fact that the rates of COVID-19 testing at the time the data were collected were lower in the UK than in many other countries, we did not analysis separately confirmed and suspected COVID-19 diagnosis and its association between COVID-19-related measures, as the prevalence of laboratory confirmed cases is likely to be underrepresented. Our analysis focused on mainly the groups of different tobacco use patterns (i.e., exclusive cigarette or e-cigarettes users, dual users or non-users) within cohorts at the time of the survey, and did not investigate the association with either the frequency of tobacco product use or the severity of COVID-19-related symptoms due to the unavailability of the data, but this is an important question for subsequent research. Furthermore, as all surveys, the analysis is limited by the fact that tobacco use was based on self-reported assessments, which makes findings at risk of misclassification and response bias leading to the underestimation of tobacco use. Therefore, tobacco use status might not be well recorded to avoid reporting bias. However, participants data on severity of symptoms, as well as asymptomatic respondents cannot be ascertained. Moreover, participant interpretation of adherence to social distancing may reflect variable understanding of what constitutes full adherence to social distancing, particularly as the study was conducted early-on in the pandemic, and therefore there is risk of misclassification bias. We attempted to mediate this, however, by making criteria for compliance with social distancing strict (only a score of 10 on a scale of 0 to 10). Lastly, due to the analytical approach and the nature of the data, we were unable to identify causal associations. To this end, this study does not examine possible mediational pathways to explain the associations observed. For instance, it is possible that the relationship between dual use and self-reported COVID-19 infection is mediated by lower compliance with social distancing, rather than as a potential direct causal relationship. Further studies should use longitudinal data to capture temporality and causal relationships, including the examination of mediators and moderators.

## 5. Conclusions

Our findings from nationally representative birth cohorts highlight dual users’ higher prevalence of having COVID-19 symptoms, self-reported infection and incompliance of social distancing behaviors. Significant associations of COVID-19 infection with dual product use, and that dual users as well as exclusive cigarette users were linked to poor adherence to social distancing behaviors. As mounting evidence suggests tobacco product use is strongly associated with COVID-19 infections and progressions, smoking cessation support and further monitoring on multiple tobacco use should be reinforced as preventive measures to tackle the pandemic.

## Figures and Tables

**Table:** **A**

	Weighted Percentages (%) with 95% Confidence Interval														
	Millennium Cohort Study (MCS), Born in 2000–2002 (Aged 18–20) (N = 2403)					Next Steps (NEXT), Born in 1989–1990 (Aged 31) (N = 1780)					British Cohort Study (BCS70), Born in 1970 (Aged 51) (N = 4022)				
	Non-Users (N = 1983)	Cigarette (N = 274)	E-cigarette (N = 65)	Dual user (N = 81)	*p*-Value †	Non-Users (N = 1477)	Cigarette (N = 182)	E-cigarette (N = 74)	Dual user (N = 47)	*p*-Value †	Non-Users (N = 3330)	Cigarette (N = 398)	E-cigarette (N = 203)	Dual user (N = 91)	*p*-Value †
Overall	78.0 (74.9–80.8)	13.7 (11.6–16.1)	3.5 (2.5–5.1)	4.7 (3.4–6.5)		75.5 (71.7–79.0)	14.3 (11.5–17.6)	5.8 (4.0–8.2)	4.4 (2.8–6.8)		75.5 (74.1–76.8)	15.2 (14.2–16.4)	6.5 (5.8–7.3)	2.8 (2.3–3.3)	
**Participant characteristics**															
Gender															
Male	48.0 (44.4–51.6)	50.7 (42.3–59.1)	51.1 (33.1–68.8)	64.8 (49.7–77.4)	0.001	44.3 (40.0–48.6)	35.6 (25.8–46.7)	47.0 (29.7–65.0)	61.6 (40.1–79.4)	0.14	49.1 (47.4–50.8)	53.7 (48.7–58.5)	63.9 (57.0–70.2)	44.84 (34.9–55.2)	<0.01
Female	52.0 (48.4–55.6)	49.3 (40.9–57.7)	48.9 (31.2–66.9)	35.2 (22.6–50.3)		55.7 (51.4–60.0)	64.5 (53.3–74.2)	53.0 (35–70.3)	38.4 (20.6–59.9)		50.9 (49.2–52.6)	46.3 (41.5–51.3)	36.1 (29.8–43.0)	55.16 (44.8–65.1)	
Socioeconomic Class ^a^															
High	4.0 (2.4–6.5)	5.3 (1.4–14.4)	4.52 (0.9–13.7)	6.1 (2.1–14.5)	<0.001	54.8 (50.7–58.8)	29.8 (21.2–40.1)	34.4 (21.2–50.5)	44.0 (24.1–66.0)	0.00	44.0 (42.3–45.7)	29.1 (24.9–33.8)	28.7 (22.8–35.3)	18.0 (11.3–27.3)	<0.001
Intermediate	6.8 (5.4–8.45)	8.4 (5.1–13.4)	10.9 (3.6–26.1)	3.7 (0.5–12.2		19.3 (16.3–22.7)	24.5 (14.6–38.0)	33.2 (17.1–54.4)	12.8 (5.1–27.3)		20.7 (19.3–22.1)	14.8 (11.7–18.7)	14.8 (10.5–20.4)	15.9 (9.6–25.0)	
Lower	18.4 (15.3–22.0)	32.9 (25.9–40.7)	29.8 (16.0–48.5)	33.6 (20.7–49.5)		14.3 (11.6–17.4)	23.0 (15.3–33.0)	23.2 (10.4–43.4)	32.3 (15–56.2)		19.09 (17.8–20.5)	33.4 (28.9–38.2)	31.3 (25.2–38.0)	38.1 (28.7–48.5)	
Not classifiable §	70.9 (66.5–74.8)	53.5 (45.6–61.2)	54.8 (36.5–71.9)	56.6 (39.7–72.1)		11.7 (9.5–14.3)	22.7 (14.4–33.9)	9.2 (2.7–23.4)	10.9 (3.5–26.3)		16.23 (15–17.5)	22.7 (18.8–27.1)	25.3 (19.8–31.7)	28.1 (19.8–38.2)	
Self-rated Health															
Excellent	17.1 (14.7–19.6)	4.5 (2.3–8.5)	16.0 (7.3–30.9)	5.6 (0.7–18.4)	<0.001	17.6 (15.0–20.7)	6.85 (1.9–18.3)	8.1 (3.3–17.2)	16.3 (3.2–45.8)	0.00	15.8 (14.6–17.1)	6.0 (4.0–8.8)	7.0 (4.1–11.5)	4.9 (1.7–11.9)	<0.001
Very good	43.3 (39.6–47.1)	32.7 (25.8–40.4)	47.2 (28.5–66.8)	28.6 (17.4–43.2)		47.9 (43.8–52.0)	36.7 (26.2–48.6)	64.1 (47.9–77.7)	57.0 (35.3–76.4)		39.2 (37.5–40.8)	30.9 (26.5–35.6)	24.4 (19.0–30.8)	24.1 (16.3–34.0)	
Good	31.4 (27.8–35.3)	42.8 (34.8–51.2)	29.4 (16.3–46.9)	47.4 (31.9–63.4)		26.6 (23.1–30.4)	38.6 (28.0–50.4)	19.6 (10.5–33.5)	19.56 (9.1–36.6)		31.2 (29.7–32.8)	39.5 (34.8–44.4)	48.2 (41.4–55.1)	38.9 (29.4–49.3)	
Fair	7.5 (5.6–10.1)	18.0 (12.3–25.5)	5.2 (2.0–11.8)	17.3 (6.5–37.2)		7.3 (5.3–9.9)	17.1 (8.9–29.9)	6.8 (1.9–17.9)	6.7 (1.6–18.7)		10.5 (9.5–11.6)	18.2 (14.7–22.3)	17.7 (13.0–23.6)	22.2 (14.7–32.0)	
Poor	0.7 (0.4–1.3)	2.0 (0.5–5.9)	2.2 (0.7–12.3)	1.1 (0.4–6.4)		0.5 (0.2–1.1)	0.8 (0.2–2.6)	1.4 (0.2–4.5)	0.5 (0.2–3.1)		3.3 (2.8–4.0)	5.5 (3.6–8.3)	2.8 (1.1–6.2)	9.9 (5.1–18.1)	
Chronic illness ^b^															
Yes	29.3 (26.4–32.4)	38.8 (31.5–46.5)	55.2 (37.1–72.0)	35.4 (20–54.5)	<0.001	32.1 (28.7–35.7)	58.1 (46.4–68.9)	40.3 (24.2–58.8)	35.4 (19.0–56.1)	<0.01	49.2 (47.5–51.0)	57.8 (52.9–62.6)	61.9 (55.0–68.4)	63.4 (53.0–72.7)	0.001
No	70.7 (67.6–73.6)	61.2 (53.5–68.5)	44.8 (28.0–62.9	64.6 (45.5–80)		67.9 (64.3–71.4)	41.9 (31.1–53.6)	59.7 (41.2–75.8)	64.6 (43.9–81.0)		50.8 (49.1–52.5)	42.2 (37.4–47.2)	38.1 (31.6–45.0)	36.6 (27.3–47.0)	
**COVID-19–related outcomes**															
Social-distancing ^c^															
Yes	N = 1017 50.4 (46.4–54.3)	N = 97 35.7 (28.3–43.9)	N = 22 37.1 (19.7–58.7)	N = 19 24.1 (12.0–42.1)	<0.001	N = 703 48.9 (44.8–53.0)	N = 73 33.6 (24.0–44.8)	N = 32 42.5 (26.1–60.7)	N = 19 38.2 (20.0–60.4)	0.18	N = 1832 54.8 (53.1–56.5)	N = 201 50.7 (45.8–55.6)	N = 113 58.0 (51.1–64.7)	N = 46 52.9 (42.6–62.9)	0.29
No	N = 965 49.6 (45.7–53.6)	N = 177 64.3 (56.1–71.7)	N = 43 62.9 (41.3–80.4	N = 62 76.0 (57.9–88.0)		N = 769 51.2 (47.0–55.2)	N = 109 66.4 (55.2–76.0)	N = 42 57.5 (39.3–73.9)	N = 28 61.8 (39.6–80.0)		N = 1493 45.2 (43.5–46.9)	N = 197 49.3 (44.4–54.2)	N = 90 42.0 (35.4–48.9)	N = 45 47.1 (37.1–57.4)	
Covid-19 Symptoms ^d^															
Yes	N = 549 27.0 (24.1–30.1)	N = 91 29.9 (23.8–36.8)	N = 24 38.8 (21.2–59.9)	N = 28 24.5 (14.5–38.0)	0.03	N = 363 24.0 (20.5–27.8)	N = 52 34.5 (23.8–47.1)	N = 21 23.3 (12.6–38.8)	N = 15 24.0 (11.3–43.3)	0.85	N = 818 26.9 (25.4–28.5)	N = 108 28.0 (23.8–32.7)	N = 66 31.6 (25.6–38.3)	N = 23 36.2 (27.0–46.6)	0.79
No	N = 1434 73.0 (69.9–75.9)	N = 183 70.2 (63.3–76.2)	N = 41 61.2 (40.1–78.8)	N = 53 75.5 (62.0–85.5)		N = 1114 76.0 (72.2–79.5)	N = 130 65.5 (52.9–76.2)	N = 53 76.7 (61.2–87.4)	N = 32 76.0 (56.7–88.7)		N = 2512 73.1 (71.6–74.6)	N = 290 72.0 (67.3–76.2)	N = 137 68.4 (61.7–74.4)	N = 68 63.8 (53.4–73.0)	
Covid-19 test ^e^															
Yes	N = 35 1.8 (1.1–2.9)	N = 4 3.9 (0.4–13.7)	N = 1 10.0 (4.4–20.3)	N = 3 2.9 (0.2–12.4)	0.60	N = 55 4.2 (2.7–6.5)	N = 8 2.7 (1.1–5.9)	N = 1 3.2 (0.8–17.4)	N = 0 ―	0.36	N = 101 3.3 (2.7–4.0)	N = 13 3.0 (1.7–5.2)	N = 4 1.3 (0.2–4.2)	N = 5 12.3 (6.8–20.8)	0.45
No	N = 1946 98.2 (97.1–98.9)	N = 270 96.1 (86.3–99.6)	N = 64 99.8 (99.0–99.9)	N = 78 97.1 (87.6–99.9)		N = 1420 95.8 (93.5–97.3)	N = 173 97.3 (94.1–98.9)	N = 73 96.8 (82.6–99.8)	N = 46 100 (―)		N = 3219 96.7 (96.0–97.3)	N = 383 97.0 (94.8–98.4)	N = 198 98.7 (95.8–99.8)	N = 86 87.8 (79.2–93.2)	
Covid-19 infection ^f^															
Yes	N = 111 5.1 (3.91–6.7)	N = 22 6.9 (3.8–11.8)	N = 9 11.4 (4.7–23.7)	N = 9 10.0 (4.4–20.3)	<0.01	N = 160 10.9 (8.6–13.8)	N = 18 9.0 (3.6–19.4)	N = 6 11.9 (1.8–37.4)	N = 7 20.4 (6.2–47.5)	0.68	N = 298 9.8 (8.8–10.8)	N = 35 8.1 (5.8–11.2)	N = 18 6.3 (3.6–10.7)	N = 11 17.5 (10.9–26.8)	0.78
No	N = 1872 94.9 (93.3–96.1)	N = 252 93.2 (88.1–96.1)	N = 56 88.6 (76.3–95.3	N = 72 90.0 (79.7–95.6)		N = 1317 89.1 (86.2–91.4)	N = 164 91.0 (80.6–96.4)	N = 68 88.1 (62.6–98.2)	N = 40 79.6 (52.5–93.9)		N = 3031 90.2 (89.2–91.2)	N = 363 91.9 (88.8–94.3)	N = 185 93.7 (89.3–96.4)	N = 80 82.5 (73.3–89.1)	

**Table:** **B**

	Weighted Percentages (%) with 95% Confidence Interval									
	National Child Development Study (NCDS), Born in 1958 (Aged 63)					Overall				
	(N = 4872)					(N = 13,077)				
	Non-Users	Cigarette	E-cigarette	Dual User	*p*-Value †	Non-Users	Cigarette	E-cigarette	Dual User	CMH χ2 ^††^
	(N = 4313)	(N = 344)	(N = 147)	(N = 68)		(N = 11,103)	(N = 1198)	(N = 489)	(N = 287)	
Overall	85.2	9.1	3.9	1.7		79.6	12.6	4.9	3	
	(84.2–86.2)	(8.4–10.0)	(3.4–4.5)	(1.4–2.2)		(78.9–80.3)	(12.0–13.2)	(4.5–5.3)	(2.7–3.3)	
**Participant characteristics**										
Gender										
Male	50.2	55.2	51.9	47	0.69	48.7	50.6	55.8	54.7	0.01
	(48.7–51.7)	(49.9–60.4)	(43.9–59.8)	(35.6–58.7)		(47.8–49.6)	(47.8–53.5)	(51.4–60.2)	(48.9–60.4)	
Female	49.8	44.8	48.1	53.1		51.3	49.4	44.2	45.3	
	(48.3–51.3)	(39.6–50.1)	(40.2–56.1)	(41.4–64.4)		(50.4–52.3)	(46.5–52.2)	(39.8–48.6)	(39.6–51.1)	
Socioeconomic Class ^a^										
High	24.1	13.7	8.3	15.2	<0.001	30.1	20.2	20.3	19.1	<0.001
	(22.8–25.4)	(10.4–17.8)	(4.7–14.0)	(8.4–25.8)		(29.3–31.0)	(18.0–22.6)	(17.0–24.1)	(14.9–24.1)	
Intermediate	16	13.2	12.7	10.2		16.1	14.6	16.7	10.4	
	(15.0–17.2)	(10.0–17.3)	(8.2–19.2)	(4.7–19.9)		(15.4–16.8)	(12.7–16.7)	(13.6–20.3)	(7.3–14.5)	
Lower	17.7	30.7	29	35.1		17.8	30.9	29.1	34.9	
	(16.6–18.9)	(26.0–35.8)	(22.3–36.9)	(24.8–47.0)		(17.1–18.5)	(28.4–33.6)	(25.2–33.3)	(29.6–40.6)	
Not classifiable §	42.2	42.4	49.9	39.5		36	34.3	34	35.6	
	(40.7–43.6)	(37.2–47.7)	(42.0–57.9)	(28.8–51.4)		(35.1–36.9)	(31.7–37.1)	(29.9–38.3)	(30.2–41.3)	
Self-rated Health										
Excellent	14.7	2	4.7	7.4	<0.001	15.9	4.7	7.7	8	<0.001
	(13.7–15.8)	(0.9–4.2)	(2.1–9.6)	(2.8–16.5)		(15.2–16.6)	(3.7–6.1)	(5.7–10.5)	(5.3–11.7)	
Very good	39.4	28.5	38	25.1		41.2	31.5	38	32.4	
	(37.9–40.8)	(23.9–33.5)	(30.6–46.1)	(16.2–36.6)		(40.2–42.1)	(28.9–34.2)	(33.8–42.4)	(27.2–38.0)	
Good	32.2	37.9	37.2	33.6		31	39.6	37.7	36.4	
	(30.8–33.6)	(32.9–43.2)	(29.8–45.2)	(23.5–45.5)		(30.2–31.9)	(36.9–42.4)	(33.5–42.1)	(31.0–42.1)	
Fair	11.4	22.5	14.1	31.3		9.9	19.1	13.1	19.6	
	(10.5–12.4)	(18.4–27.3)	(9.3–20.7)	(21.5–43.1)		(9.3–10.5)	(17.0–21.5)	(10.4–16.4)	(15.3–24.6)	
Poor	2.4	9.1	6	2.6		2.1	5	3.4	3.8	
	(1.9–2.9)	(6.4–12.7)	(3.0–11.2)	(0.1–10.3)		(1.8–2.4)	(3.9–6.4)	(2.1–5.5)	(2.0–6.8)	
Chronic illness ^b^										
Yes	62.1	69	66.9	66.6	0.48	48.6	57.1	58.9	50.2	<0.001
	(60.6–63.6)	(63.9–73.7)	(58.9–74.1)	(54.7–76.7)		(47.6–49.5)	(54.2–59.9)	(54.5–63.2)	(44.4–56.1)	
No	37.9	31	33.1	33.4		51.5	42.9	41.1	49.8	
	(36.4–39.4)	(26.3–36.2)	(25.9–41.1)	(23–45.3)		(50.5–52.4)	(40.1–45.8)	(36.8–45.5)	(43.9–55.6)	
**COVID-19–related outcomes**										
Social-distancing ^c^										
Yes	N = 2700	N = 204	N = 86	N = 45	0.43	N = 6260	N = 575	N = 253	N = 129	<0.001
	64	61.8	52.1	60		56.9	47.9	50.9	42.8	
	(62.6–65.4)	(56.5–66.8)	(44.1–60.0)	(48.1–70.8)		(55.9–57.8)	(45.1–50.8)	(46.4–55.3)	(37.2-48.6)	
No	N = 1606	N = 139	N = 61	N = 23		N = 4843	N = 623	N = 236	N=158	
	36	38.2	47.9	40		43.1	52.1	49.1	57.2	
	(34.6–37.5)	(33.2–43.5)	(40.0–56.0)	(29.2–51.9)		(42.2–44.1)	(49.2–54.9)	(44.7–53.6)	(51.4-62.8)	
Covid-19 Symptoms ^d^										
Yes	N = 946	N = 89	N = 31	N = 22	0.08	N = 2676	N = 340	N = 142	N = 88	0.02
	23.1	25.7	20.6	41.4		25	28.8	28	31.3	
	(21.8–24.3)	(21.3–30.6)	(14.8–27.9)	(30.4–53.3)		(24.2–25.8)	(26.3–31.4)	(24.2–32.1)	(26.2–36.9)	
No	N = 3367	N = 255	N = 116	N = 46		N = 8427	N = 858	N = 347	N = 199	
	77	74.3	79.4	58.6		75	71.2	72	68.7	
	(75.7–78.2)	(69.4–78.7)	(72.01–85.2)	(46.8–69.6)		(74.2–75.8)	(68.6–73.7)	(67.9–75.8)	(63.1–73.8)	
Covid-19 test ^e^										
Yes	N = 81	N = 5	N = 3	N = 3	0.45	N = 288	N = 30	N = 11	N = 12	0.73
	2.7	0.8	1.4	7.5		2.9	2.6	1.5	5.9	
	(2.3–3.3)	(0.1–2.6)	(0.1–5.2)	(2.9–16.6)		(2.6–3.3)	(1.8–3.6)	(0.7–3.03)	(3.7–9.4)	
No	N = 4210	N = 335	N = 143	N = 65		N = 10,815	N = 1168	N = 478	N = 275	
	97.3	99.2	98.6	92.5		97.1	97.4	98.5	94.1	
	(96.7–97.7)	(97.4–99.9)	(94.8–99.9)	(83.4–97.1)		(96.8–97.4)	(96.4–98.2)	(96.9–99.4)	(90.6–96.3)	
Covid-19 infection ^f^										
Yes	N = 244	N = 16	N = 9	N = 6	0.57	N = 815	N = 91	N = 42	N = 33	0.04
	5.7	3.8	4.2	12.9		7.5	6.8	7.3	14.8	
	(5.1–6.5)	(2.1–6.4)	(1.8–8.9)	(6.7–23.1)		(7.0–8.0)	(5.5–8.4)	(5.3–10.0)	(11.2–19.5)	
No	N = 4068	N = 328	N = 138	N = 62		N = 10,288	N = 1107	N = 447	N = 254	
	94.3	96.2	95.8	87.1		92.6	93.2	92.7	85.2	
	(93.6–95.0)	(93.6–97.9)	(91.1–98.2)	(76.9–93.4)		(92.0–93.03)	(91.6–94.5)	(90.0–94.7)	(80.5–88.9)	

Note: 95% confidence intervals associated with weighted proportions were calculated using the Agresti-Coull method, percentages may not always equal 100% due to rounding; N indicates unweighted counts of the number of cases for each cell. MCS: refers to the Millennium Cohort Study, born in 2000–2002 (aged 18–20); NEXT: refers to Next Steps, born in 1989-1990 (aged 31); BCS70: refers to British Cohort Study, born in 1970 (aged 51); NCDS: refers to National Child Development Study, born in 1958 (aged 63). †: χ2 test within cohorts; ††: CMH, Cochran–Mantel–Haenszel χ2 test controlling for cohorts (tests association between participant characteristics, COVID-19–related risk factors and different smoking patterns controlling for cohorts); ―: No observations. a: socioeconomic class, the 2010 National Statistics Socioeconomic Analytical Classes (NS-SEC) prior to the COVID-19 outbreak. (Higher: higher managerial, administrative and professional occupations; Intermediate: intermediate occupations, small employers and own account workers; Lower: routine and manual occupations). b: chronic illness, suffering from a long-standing illness, such as: cancer, cystic fibrosis, asthma, Chronic Obstructive Pulmonary Disease, bronchitis, diabetes, recurrent back pain other back problems, hearing problems, high blood pressure, heart diseases, depression or other psychiatric problems, obesity, AIDS/HIV or neurological disorders. c: covid-19 Symptoms, experiencing symptoms such as: dry cough, fever, loss of sense of smell or taste, or shortness of breath in the past 2 weeks. d: social-distancing, extent of social distancing compliance: either fully compliant or not. e: Covid-19 diagnosis, either having confirmed/suspected Covid-19 diagnosis, or uninfected. f: Covid-19 test, either received a Covid-19 test or not.

**Table 2:** Associations between COVID-19–related measures and exclusive or dual use of cigarette and e-cigarettes.

	Adjusted Odds Ratio (aOR) with 95% Confidence Interval		
	aOR	95% CI	*p*-Value
Experiencing Covid-19 Symptoms ^a^ (N = 12,804)			
Non-smokers	Ref.		
Cigarette	1.21	0.87–1.69	0.26
E-cigarette	1.08	0.61–1.89	0.8
Dual Use	1.41	0.79–2.54	0.25
Adhered to Social-distancing ^b^ (N = 12,785)			
Non-smokers	Ref.		
Cigarette	0.72	0.63–0.92	<0.01
E-cigarette	0.82	0.62–1.09	0.17
Dual Use	0.58	0.41–0.83	<0.01
Confirmed/Suspected Covid-19 diagnosis ^c^ (N = 12,803)			
Non-smokers	Ref.		
Cigarette	1.1	0.89–1.36	0.66
E-cigarette	1.22	0.90–1.65	0.97
Dual Use	2.15	1.15–4.05	0.02
Received Covid-19 test ^d^ (N = 12,785)			
Non-smokers	Ref.		
Cigarette	1.05	0.75–1.48	0.75
E-cigarette	0.85	0.48–1.50	0.16
Dual Use	1.97	0.62–6.32	0.25

Note: multivariable logistic regression models adjusted for sex, socioeconomic class, self-rated health, chronic illness and cohorts. aOR: adjusted odds ratio. ^a^: covid-19 Symptoms, experiencing symptoms such as: dry cough, fever, loss of sense of smell or taste, or shortness of breath in the past two weeks. ^b^: social-distancing, extent of social distancing compliance: either fully compliant or not. ^c^: Covid-19 diagnosis, either having confirmed/suspected Covid-19 diagnosis, or uninfected. ^d^: Covid-19 test, either received a Covid-19 test or not.

## Data Availability

The datasets generated and analysed in the current study are available from the UK Data Service. (https://beta.ukdataservice.ac.uk/datacatalogue/studies/study?id=8658 (accessed on 10 December 2020)).
